# A Water Balloon as an Innovative Energy Storage Medium

**DOI:** 10.3390/polym14163396

**Published:** 2022-08-19

**Authors:** Chun-Ti Chang, Pin Tuan Huang

**Affiliations:** Department of Mechanical Engineering, National Taiwan University, Taipei 10617, Taiwan

**Keywords:** balloon, energy storage, Gough–Joule effect, waste heat recycle

## Abstract

Soft rubbery materials are capable of withstanding large deformation, and stretched rubber contracts when heated. Additionally, rubber balloons exhibit non-monotonic pressure–volume curves. These unique properties have inspired numerous ingenious inventions based on rubber balloons. To the authors’ knowledge, however, it is surprising that these properties have not inspired any study that exploits the elasticity of rubber balloons for energy storage. Motivated by these, this study examines the performance of water balloons as energy storage media. In each experiment, a single water balloon is implemented using a flat membrane, and it is subject to repeated inflation, heating, deflation, and cooling. Inflating the balloon deposits energy into it. The heating simulates the recycling of waste heat. The balloon delivers work during its deflation. Finally, the cooling completes the energy-storage cycle. The performance is evaluated in terms of the balloon’s transferred energies, efficiencies, and service life. Simple as it is, a water balloon is actually an impressively efficient energy storage medium. The efficiency is 85–90% when a water balloon stores and releases energy at room temperature. Recycling waste heat can boost a balloon’s efficiency beyond 100%, provided that the cost of the heat is negligible so that the heat is not taken as part of the input energy. However, heating shortens the service life of a balloon and reduces the total energy it can accommodate. By running fatigue tests on balloons, this study reveals the trade-off between a water balloon’s efficiency and its longevity. These results shall serve as a useful guide for implementing balloon-based mechanical devices not limited to energy-storage applications.

## 1. Introduction

Soft rubber membranes possess many attractive properties for implementing mechanical applications. The capability of withstanding large deformation is perhaps the most unique one. This property enables a rubber membrane to form a balloon and enclose a fluid with several hundred times its volume. In fact, a balloon formed by a flat membrane is known to possess a non-monotonic relation between its pressure *P* and its volume *V* [1,2,3,4]. The non-monotonicity of the pressure–volume (P–V) curve incurs snap-through and snap-back instabilities [5,6,7]. These properties enable a rubber membrane to exert forces with a large displacement volume and/or a fast response. In published works, the properties have been exploited to implement pumps [8,9,10,11] for flow control, catheters [12,13,14,15,16] for angioplasty [17], artificial muscles for robots [18,19,20], harvesters for renewable energy [21,22,23], and elastocaloric heat pumps for thermal energy management [24]. When a pressurized fluid inflates a rubber membrane into a balloon, the membrane stores the work done by the fluid as its elastic potential energy. The energy can be withdrawn for utility by deflating the balloon and releasing the pressurized fluid. The inflation and deflation of a balloon are analogous to the charge and discharge of a battery. Motivated by these, this study develops water balloon energy storage (WBES), in which a water balloon functions as an energy storage medium to accommodate the energy transported by subcooled liquid water. A first scientific question here is the long-term evolution of the energies associated with the repeated inflation and deflation of a water balloon. Additionally, a second scientific question is the influence of heat upon these energies and the balloon’s durability. To address these questions, this study experimentally evaluates the performance of WBES, focusing on a single balloon’s thermo-mechanical responses when it is subject to cyclic thermal and mechanical loads. To the authors’ knowledge, this is the first study that proposes an energy-storage technology using a rubber balloon’s thermo-mechanical properties.

Naive and playful as it seems, WBES may be an excellent complement to relevant existing technologies. The greatest advantage of WBES is its simplicity. As reported later, a WBES system can be as simple as a solid tube with a clamped membrane on one end and a source of pressurized water on the other. Such a simple setup leads to several advantages over relevant existing technologies. For example, WBES does not cause the severe environmental impact of pumped hydro energy storage as it does not require large-scale modification of the environment. Additionally, WBES does not suffer from the same geological constraints of underground compressed-air energy storage (CAES) [25]. Neither does it involve the complexity of anchoring large structures deep under water as underwater CAES does [26,27,28]. Being a purely mechanical technology, WBES does not incur the cost, labor, or risk of recycling the electrochemical waste for lithium batteries [29,30]. WBES and flywheel energy storage (FES) are similar in terms of storing mechanical energy. Unlike FES, however, WBES stores mechanical energy in the form of elastic potential and does not involve any high-speed solid motion. This eliminates the risk of a catastrophic failure at the end of the system’s service life, thus saving the cost of “bunkering” the system [31]. As reported later, WBES does suffer from a stand-by loss similar to those of lithium-ion batteries and FES [31,32,33]. Unlike these technologies, however, WBES can make up for its own stand-by loss by recycling low-grade waste heat. In fact, the heat pertains to such low grade that its optimal temperature is around 30–40 °C. To the authors’ knowledge, no existing energy storage technology is capable of utilizing such low-grade heat. Again, WBES stores energy only in the form of a membrane’s elastic potential, and the water experiences no phase change even when the balloon recycles waste heat. These requirements restrict WBES to operate within tens of degrees Celsius around room temperature. With the range of operating temperatures, WBES imposes virtually no constraint on the thermal durability of its mechanical parts, not to mention the complexities of handling fluids at extreme temperatures such as in the case of liquid-air energy storage [34,35,36,37,38,39,40,41,42]. Based on these, WBES is expected to complement existing energy-storage technologies by serving as an alternative that is simple, safe, clean, flexible in site selection, and capable of recycling low-grade waste heat.

Regarding how WBES recycles waste heat, a viable solution is provided by the Gough–Joule effect, the tendency of a stretched rubber to become more stressed when heated [43,44]. The effect has been exploited in the development of rubber heat engines [45,46,47]. Despite their simple structures, these engines can produce work out of temperature differences as small as 30°C [48,49,50,51]. Motivated by these pioneering works, this study exploits the Gough–Joule effect to boost the pressures of balloons by heating them after their inflations. As reported later, heating helps a balloon keep its pressure during its deflation. This compensates for the stand-by loss due to the membrane’s relaxation and helps to recover more of the work deposited during the inflation.

Recycling waste heat with a balloon reduces the balloon’s service life. This is due to the influence of heat on the strain-induced crystallization (SIC) of rubber. Natural rubber is known to crystallize when it is stretched [52,53,54,55,56,57]. When a rubber with a crack is stretched, SIC transforms the molecules near the crack tip into crystallites [53,55], thereby retarding crack growth. SIC thus endows natural rubbers with a strength superior to that of its synthetic counterpart, synthetic polyisoprene rubber, which is not crystallizable [54]. Heating retards SIC and melts the crystallites of rubber [52,53,56,57]. Consequently, heating a stretched rubber weakens the rubber and shortens its service life [56,57,58]. While heating a balloon compensates for the energy loss due to the membrane’s relaxation, it also makes the balloon more vulnerable to crack growth. Accordingly, adopting the Gough–Joule effect for energy storage incurs a trade-off between the balloon’s longevity and efficiency of energy storage.

This study tests the performance of a single water balloon as an energy storage medium. In each experiment, a balloon is subject to cyclic mechanical and thermal loads. The performance shall be quantified in terms of the balloon’s transferred energies, efficiencies, and service life. Instead of using compressed air, this study adopts subcooled liquid water as the working fluid for its higher density and its incompressibility. For fluid streams with the same volume flow rate, the one with a higher density can deliver a larger inertia force for work production. On the other hand, the incompressibility enables precise and repeatable prescription of the balloon’s volume that is immune to the disturbance associated with any temperature fluctuation. A single balloon accommodates only a limited amount of energy. For practical utility, the capacity can be scaled up by implementing a large number of balloons in arrays. Working with multiple interconnected balloons requires suitable control of the scavenging between the balloons [59]. For simplicity, this study focuses on the behavior of one single balloon and saves the multi-balloon problem for a future study. In what follows, Section 2 reports the operation and performance evaluation of WBES, and Section 3 presents all results and analyses, followed by the conclusion in Section 4.

## 2. Materials and Methods

This section reports the operation of WBES and the methods for testing its performance. In what follows, Section 2.1 proposes the energy-storage cycle of WBES, its synchronization with daily power demand, and the candidate power sources for its operation. Subsequently, Section 2.2 presents the experimental setup for testing the performance of WBES, and Section 2.3 defines the parameters for performance evaluation.

### 2.1. Energy-Storage Cycle

**Figure 1:**
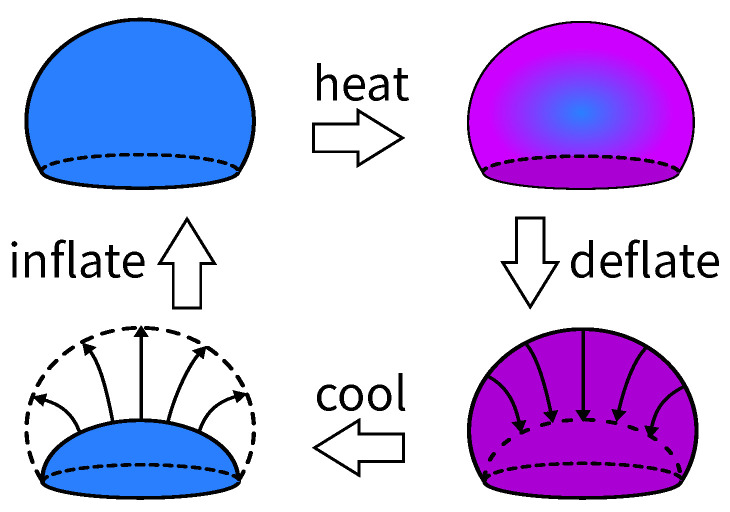
(Color online) A schematic illustration of the cycle for water balloon energy storage (WBES).

Much like other energy storage media, a water balloon operates in a cycle to store and supply energy. Figure 1 illustrates the processes of the cycle, in which a balloon is inflated with cold water, heated, deflated when it is warm, and cooled when it is flat. The inflation process deposits energy into the balloon, and the balloon’s membrane transforms the work done by the water into its elastic potential energy. The heating process simulates the recycling of waste heat by heating the balloon to boost its pressure. This can make up for a substantial proportion of the pressure drop due to the membrane’s relaxation. Subsequently, the balloon releases pressurized water to propel a water turbine (not shown) and produce work during the deflation process. Finally, the cooling process completes the cycle by bringing the balloon’s membrane back to the initial (i.e., pre-inflation) temperature.

The processes in Figure 1 can be readily synchronized with the daily demand for power in cities. The demand is typically higher during the day and lower at night. As such, the balloon should be inflated at night and heated with solar heat or other waste heat during the day. Then the balloon can release its pressurized water for power generation when energy is most needed. Based on this, a water balloon repeats the cycle in Figure 1 once every day.

WBES accommodates the energy of the pressurized water, and the water may be pumped from different sources. One intuitive option is to pump the water mechanically with the electricity during the off-peak hours or from a renewable source. Alternatively, a WBES system can be integrated with a rainwater-harvesting (RWH) system [60,61,62]. This can be implemented, for example, by installing the WBES system in the basement of a building and connecting it either directly to the gutter through the downpipes or a header tank right below the roof [62]. In that case, WBES stores the gravitational energy that the building retains in the rain. This integrates WBES and the rainwater harvester into an eco-friendly energy harvesting system.

### 2.2. Experimental Setup

**Figure 2:**
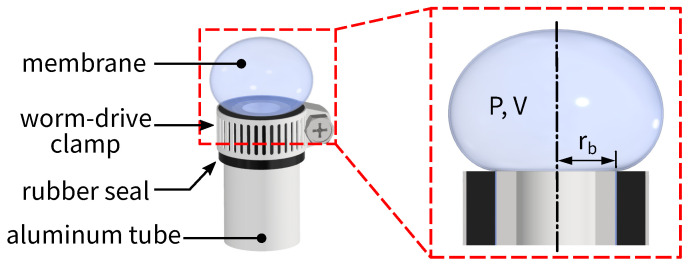
(Color online) A schematic illustration of an inflated balloon with pressure *P*, volume *V*, and base radius $r_{b}$ in this study. The balloon is prepared by clamping a flat piece of rubber membrane around the end of an aluminum tube and injecting de-ionized water from inside the tube.

For each experiment in this study, a balloon is prepared by clamping a membrane (model 8166NS, by Cranberry International Sdn Bhd, Shah Alam, Malaysia) around the end of a circular tube, as shown in Figure 2. The material of the membrane is natural rubber vulcanized with zinc oxide, sulfur, ZDEC, ZDBC, antioxidant, 20% Aerodisp W7520, peppermint oil, and spearmint oil according to the manufacturer. The membrane itself is originally flat and has a uniform thickness of h=216±4 μm. It is clamped around the tube with a worm-drive clamp, and a rubber seal is sandwiched between the clamp and the membrane to prevent leakage. The radius of the circular tube is 8.3 mm, which is the balloon’s base radius $r_{b}$. Once assembled, a balloon is degassed in a water tank and kept there to be connected with other mechanical parts for the experiment.

**Figure 3:**
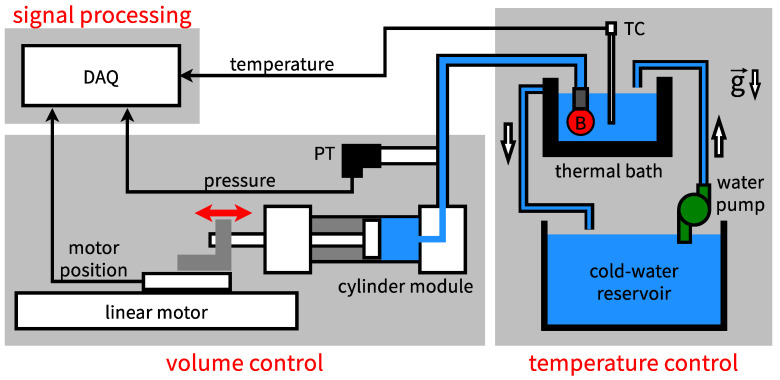
(Color online) The experimental setup for testing a water balloon with cyclic mechanical and thermal loads. DAQ: data acquisition system. PT: pressure transducer. TC: thermocouple. B: balloon.

Once prepared, a balloon is tested with the setup in Figure 3. The setup consists of a volume control unit, a temperature control unit, and a signal processing unit. The volume control unit applies the mechanical load to the balloon and executes the inflation and the deflation processes in Figure 1. The components are a cylinder module (MBR40-200, Chanto Air Hydraulics Co., Ltd., Taichung City, Taiwan), a linear motor (model CLMS-1010, by Chieftek Precision, Tainan City, Taiwan), a motor controller (model Will1-B-3, by Chieftek Precision, Tainan City, Taiwan), and a pressure transducer (PT, model PT-VP-2P-G4-H-S1-C-24V, by Atlantis Inc., Taipei City, Taiwan). Prior to each experiment, the cylinder module is degassed in a water tank and connected to a degassed balloon in the tank. Then the piston rod of the cylinder module is connected to the linear motor on a workbench. Subsequently, the inflation process is executed by having the cylinder module extrude water into the balloon. This stores energy in the balloon. During the deflation process, conversely, the cylinder module withdraws the water from the balloon. This simulates the situation when the balloon releases its pressurized water for work production. In both processes, the linear motor actuates the cylinder to control its displacement and speed. The controller drives the motor and keeps track of its displacement, and the motion is prescribed through an operating software (CPC GUI version 0.5.20, by Chieftek Precision, Tainan City, Taiwan). The motor’s displacement is monitored to deduce the balloon’s volume, and the pressure transducer reports the balloon’s pressure. With the volume and pressure, the energies associated with the inflation and deflation processes can be obtained through numerical integration.

A volume control unit such as the one in Figure 3 is certainly not the only way to apply mechanical load to a balloon. Alternatively, the mechanical load can be applied by prescribing the water’s pressure instead of its volume (flow rate). Practically, such pressure-controlled mechanical load can be implemented, for example, by connecting the balloon to the header tank of a rainwater harvesting system, see Section 2.1. However, balloons are known to possess non-monotonic P–V curves. Where the pressure and the volume do not increase with one another (a P–V curve’s *descending segment*), the pressure-controlled mechanical load incurs the snap-through instability during inflation and the snap-back instability during deflation. These instabilities, in return, prohibit access to the balloon’s pressure and volume at least over the descending segment. The pressure-controlled mechanical load thus makes a complete P–V curve unavailable and evaluating transferred energies impossible. In contrast, the volume control unit in Figure 3 prescribes the water’s volume (flow rate) and measures the balloon’s pressure. The resulting mechanical load is volume-controlled and incurs neither the snap-through nor the snap-back instability, thus making the entire P–V curve accessible through direct measurement. Because of these, the volume-controlled mechanical load is preferred and has been adopted in this study.

The temperature control unit applies the thermal load to the balloon and manages the heating and the cooling processes of Figure 1. This unit consists of the thermal bath, the water pump, the cold-water reservoir, and the thermocouple (TC) in Figure 3. The thermal bath is a small aluminum container with ten embedded heaters (not shown). It is filled with tap water at room temperature prior to each experiment. The balloon is immersed in the bath throughout the experiment. The temperature of the bath is monitored through the thermocouple. During the heating process, the heaters are turned on to raise the temperature of the water in the bath. This heats up the balloon. During the cooling process, the water pump delivers water at room temperature from the reservoir to chill the (hot) water in the bath. On the side wall of the bath, an overflow orifice is implemented to guide excess water back to the reservoir, thereby keeping a constant water level in the bath.

The signal processing unit in Figure 3 serves two purposes. The first purpose is to record the pressure of the balloon, the temperature of the thermal bath, and the displacement of the linear motor. The second purpose is to control the temperature of the thermal bath. These functions are implemented with a data-acquisition system (DAQ, model cDAQ-9178, NI-9203, NI-9403, NI-9212, and NI-9361, by National Instruments, Austin, TX, USA). In experiments, the signals of the pressure transducer, the thermocouple, and the linear motor are recorded by the DAQ with a 25 Hz sampling frequency. For the heating process, the DAQ turns the heaters on in the beginning. Then it keeps track of the thermocouple measurement to shut the heating power when the prescribed thermal load temperature $T_{t}$ is reached. For the cooling process, the DAQ controls the water pump through an electromagnetic relay. No data are recorded during the cooling process because the balloon is flat during that process.

**Figure 4:**
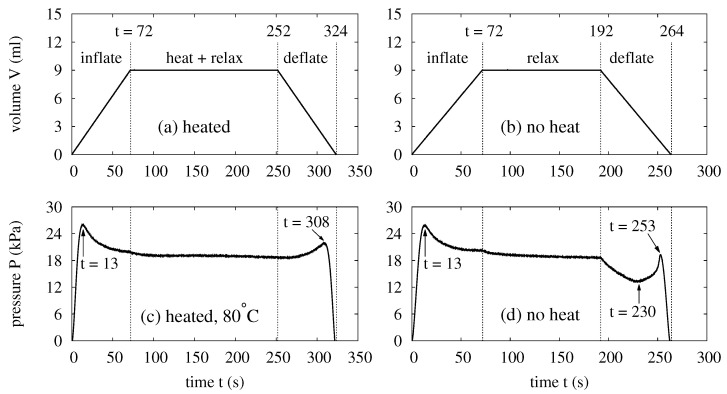
The control signals and measured responses for the balloons. (**a**) The mechanical load for the experiments with thermal load. (**b**) The mechanical load for the experiments without thermal load. (**c**) The pressure of a balloon subject to the mechanical load in (**a**) and a thermal load at $T_{t}=80{}^{\circ}$C. (**d**) The pressure of a balloon subject to the mechanical load in (**b**).

Following Figure 1, this study tests the response of a water balloon when it is subject to cyclic mechanical and thermal loads. These experiments require the collaboration of all units in Figure 3. To illustrate the experimental conditions, Figure 4 shows the mechanical load in (a) and the corresponding pressure of a balloon in (c). Starting at t=0, the mechanical load is applied by injecting 9 mL of de-ionized water at 0.126 mL/s into the balloon. The corresponding speed of the linear motor is 0.01 mm/s, and the process takes 72 s. The heating process follows the inflation to start at t=72 s, lasts for 180 s, and ends at t=252 s. As mentioned, the thermal bath is heated during the process until the prescribed thermal load temperature $T_{t}$ is reached. If $T_{t}$ is reached within 180 s (say, 120 s), the balloon is left to relax for the rest of the time (i.e., 60 s). In this study, thermal loads are applied with $T_{t}=$ 30 °C, 40 °C, 60 °C, and 80 °C.

Each heated balloon is deflated to deliver pressurized water for power generation. This is the deflation process. In Figure 4a, the deflation starts at t=252 s when the cylinder withdraws the water from the balloon at 0.126 mL/s. This is the same flow rate for the inflation. After the deflation, cold water is pumped from the reservoir into the thermal bath to cool the bath. The temperature of the reservoir is close to the room temperature, about 23 °C. The process continues for 3 min, which is sufficient to reduce the bath’s temperature back to the room temperature.

Experiments without thermal load are also conducted to reveal the effects of heating. The setup in Figure 3 is used. However, the temperature control unit is not activated, and the balloon is only subject to cyclic mechanical load when it is immersed in the thermal bath filled with water at room temperature. For these experiments, Figure 4 shows the profile of the mechanical load in (b) and the corresponding pressure of the balloon in (d). In the beginning, each balloon is inflated with 9 mL of water at 0.126 mL/s, the same flow rate as the experiments with the thermal load. The process takes 72 s. Subsequently, an inflated balloon is allowed to relax for 120 s until t=192 s. Then the balloon is deflated with the same flow rate for its inflation. The deflation takes another 72 s and terminates at t=264 s. A balloon without heating requires no cooling, and it is left to idle for only 30 s after its deflation and before its next inflation.

Each balloon in this study possesses a non-monotonic relation between its pressure and volume. As a reminder, the balloon is prepared from a clamped circular membrane that is originally flat and uniformly thick. For such a balloon, the membrane’s curvature and (nonlinear) elasticity underlie the pressure–volume relation. The membrane’s elastic modulus increases relatively slowly [63] when the balloon starts to grow in size and the stretch is relatively small. In that case, the influence of the elasticity upon the pressure is relatively minor, and the evolution of the pressure roughly follows that of the membrane’s curvature. For the balloons in Figure 4, this causes the pressure increase between t=0 s and t=13 s and the pressure drop afterwards. Further inflating the balloons raises the membranes’ elastic moduli more rapidly [63], and the influence of the elasticity becomes more significant. In Figure 4c,d, the growing influence of the elasticity makes the pressure decrease at more and more gentle rates. In fact, the pressure would start to rise if the balloons were further inflated beyond 9 mL after t=72 s. Based on the discussion thus far, the competition between the membranes’ curvature and elasticity causes the non-monotonic pressure evolution during the balloon’s inflation.

The competition between the membrane’s curvature and elasticity persists during the balloon’s deflation. As such, the pressure also evolves non-monotonically as it returns to zero. In Figure 4c, the pressure evolution during the deflation resembles the time-reversed (i.e., from t=72 s back to t=0) pressure evolution of the inflation, and the pressure is slightly lower because of the membrane’s relaxation. In Figure 4d, the balloon without heating relaxes much more, and the pressure drops significantly once the deflation starts. The pressure drop continues until t=230 s when the membrane’s curvature dominates again. Then the pressure gently rise to a maximum at t=253 s and quickly returns to zero at t=264 s.

The non-monotonic relation between the pressure and the volume is an interesting feature of the balloons. In Figure 4, the curve segments with opposite trends of pressure and volume evolutions cause the non-monotonicity by creating the descending segment in a balloon’s in a balloon’s P-V curve. The descending segment incurs the snap-through and snap-back instabilities when the mechanical load is pressure-controlled. These instabilities are attractive features of a balloon as they enable the invention of devices with fast responses [19,20,24]. For energy storage, the descending segment is also attractive because operating a balloon over this segment enables the balloon to be charged more and more easily during inflation. Additionally, it also makes the balloon deliver more and more momentum as it discharges its energy during the deflation. In this study, 9 mL is chosen as the maximum volume of each balloon to cover the descending segment of the P–V curve. Indeed, the purpose is to demonstrate the utility of these features to the advantage of energy storage.

### 2.3. Performance Indicators

In this study, a water balloon’s performance is evaluated in terms of its service life, the energy it stores during its inflation, the energy it delivers during its deflation, its round-trip efficiency in each cycle, and its average efficiency throughout its service life. All these parameters are available from the P–V curves of balloons. In what follows, the P–V curve of an inflation process shall be referred to as an *inflation curve* and that of a deflation process a *deflation curve*.

**Figure 5:**
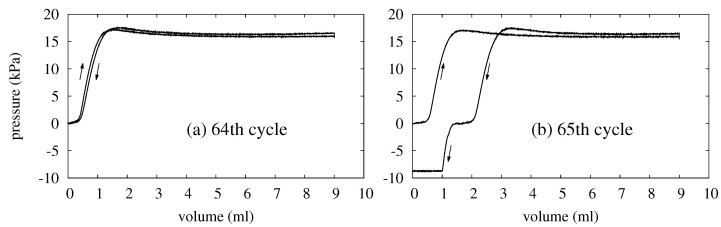
Pressure–volume (P–V) curves showing the failure of a balloon. (**a**) The P–V curves of the balloon’s 64th cycle, which is the balloon’s last cycle before it fails. (**b**) The P–V curves of the 65th cycle, which is the balloon’s first cycle with a clear sign of failure.

In each experiment, a balloon is tested until it fails. The balloon’s service life is the maximum number of energy-storage cycle $N_{\max}$ that the balloon survives, and it can be read off from the P–V curves without any numerical data analysis. As an example, Figure 5 shows the P–V curves of two consecutive cycles during which a balloon fails. In both plots, the arrows indicate the directions in which the balloon’s state evolves. An arrow pointing to the upper right means that the curve segment next to it corresponds to an inflation process. Conversely, an arrow pointing to the lower left indicates that the balloon deflates along the curve segment nearby.

The failure of a balloon is recognized when the balloon’s pressure starts to turn negative during a deflation. In Figure 5a, the balloon behaves as it has been in all previous cycles, and its pressure remains positive. The 64th cycle is the last one without a negative pressure and hence the one right before the balloon fails. In the 65th cycle, however, the pressure drops to zero as the balloon deflates but should still possess 2 mL of water. The 2 mL is apparently lost, and withdrawing that water ends up creating a suction in the balloon. This results in negative pressure, which is a clear sign of the balloon’s failure. Because the balloon operates without failure for 64 cycles, the service life of that balloon is $N_{\max}=64$ cycles.

Besides their service lives, the balloons are also examined in terms of the energies they store and release and their efficiencies of energy storage. During the *j*-th cycle, the energies that a balloon stores ($W_{ij}$) and releases ($W_{oj}$) are obtained by numerically integrating the pressure with respect to the volume as

(1) $$W_{ij}=\int_{0}^{V_{\max}}P_{ij}dV_{ij},\;W_{oj}=\int_{0}^{V_{\max}}P_{oj}dV_{oj},$$

where $V_{\max}=$ 9 mL, the first subscript specifies whether a parameter pertains to inflation (*i*) or deflation (*o*), and the second subscript *j* specifies the number of the cycle to which the parameters pertain. Throughout its service life, a balloon stores $W_{i}$ and delivers $W_{o}$ of energies in total. These total energies are obtained by summing over $W_{ij}$ and $W_{oj}$ as

(2) $$W_{i}=\sum_{j=1}^{N_{\max}}W_{ij},\;W_{o}=\sum_{j=1}^{N_{\max}}W_{oj}.$$

While $W_{ij}$ and $W_{oj}$ clearly reflect the evolution of a balloon’s capacity, they are not useful for comparing the evolutions of the energies between different balloons. This is because two balloons typically have different pressures at the same volume since the beginning of the experiments, and the energies depend on the pressures according to Equation (1). To enable the comparison, $W_{ij}$ and $W_{oj}$ are normalized by $W_{i1}$, the energy for the balloon’s first inflation,

(3) $$W_{ij}^{*}=\frac{W_{ij}}{W_{i1}},\;W_{oj}^{*}=\frac{W_{oj}}{W_{i1}}.$$

Through $W_{ij}^{*}$ and $W_{oj}^{*}$, the evolutions of $W_{ij}$ and $W_{oj}$ can be directly compared among different balloons. Finally, the round-trip efficiency $\eta_{j}$ of the *j*-th cycle is defined as

(4) $$\eta_{j}=\frac{W_{oj}}{W_{ij}},$$

and the average efficiency η of a balloon throughout its service life is

(5) $$\eta =\frac{W_{o}}{W_{i}}.$$

The definitions of the efficiencies in (4) and (5) essentially ignore the cost of the heat for boosting a balloon’s pressure. This is because low-grade waste heat is meant to be used, and the cost of the heat is negligible. As mentioned in Section 1, the temperature of the heat is only tens of degrees Celsius above the room temperature. Such waste heat is indeed freely available from the solar radiation in summer or the condenser of an air-conditioning system. Consequently, an efficiency beyond 100% is possible.

## 3. Results and Discussion

This study includes 15 experiments, each testing a single water balloon as an energy storage medium. The key parameters are presented in Table 1, where $T_{m}$ is the average and $\sigma_{T}$ is the standard deviation of the thermal bath temperature during the deflation process. All other parameters are defined in Section 2. The experiments are organized by their thermal load temperatures $T_{t}$ and their chronological orders. No thermal load is applied in experiments 1–3, and their temperatures $T_{t}$ and $T_{m}$ are specified as room temperatures (RT). In the following discussions, each balloon shall be referred to by the identity of its experiment and each thermal load by its temperature $T_{t}$. Accordingly, two or more thermal loads shall be referred to as *the same thermal load* if they are applied with the same temperatures $T_{t}$; otherwise, they are referred to as *different thermal loads*.

According to Table 1, temperature overshoot (i.e., $T_{m}>T_{t}$) is typical in the experiments. The overshoot occurs because raising the thermal bath’s temperature to $T_{t}$ requires the heaters (and perhaps the aluminum container as well) to be warmer than $T_{t}$. As such, the heaters continue to heat up the water even after their power is turned off. The overshoot could have been minimized if the heating were managed with a temperature controller. According to Table 1, however, $T_{m}$ of the same $T_{t}$ are fairly close. Additionally, $\sigma_{T}$ is less than 1.2 °C for balloons #4–#15 and less than 0.6 °C for 10 of the 12 experiments. These facts suggest that the overshoot is repeatable across different experiments. Because of this, no temperature controller is adopted for the experiments of this study.

The content of Table 1 provides an overview of the experiments. The relation between the temperatures $T_{t}$, $T_{m}$ and $\sigma_{T}$ has just been reported, and the rest of the parameters are discussed in subsequent sections. In what follows, Section 3.1 investigates the factors that influence a balloon’s service life, and Section 3.2 presents the experimental observations through the P–V curves of two selected balloons. Subsequently, Section 3.3 discusses the evolution of transferred energies, and Section 3.4 examines the efficiencies of WBES. Finally, Section 3.5 reveals an interesting scientific discovery about the permanent deformation of a flat membrane based on the experimental observations.

### 3.1. Service Life

According to Table 1, the balloons survive tens to hundreds of cycles depending on the thermal load. The balloons do possess different service lives even if they are subject to the same thermal loads. Overall, however, heating accelerates a balloon’s failure. This is manifest from the correspondence between a higher $T_{t}$ and a smaller $N_{\max}$: without any thermal load, a balloon survives several hundred cycles. With $T_{t}=30{}^{\circ}$C, $N_{\max}$ falls below 200 cycles. Further raising $T_{t}$ to 40°C leads to an $N_{\max}$ around 100 cycles, and $T_{t}=60{}^{\circ}$C reduces $N_{\max}$ to 50–80 cycles. Finally, with $T_{t}=80{}^{\circ}$C, the balloons fail in 50 cycles. As mentioned earlier, stretching a rubber induces crystallization in the rubber, and the crystallites strengthen the rubber. However, heating retards the crystallization and weakens the rubber. These effects accelerate crack growth in the rubber and eventually shorten the service life of a balloon.

The service lives of balloons in Table 1 may seem too short to adopt WBES for practical utility. For the other energy storage systems mentioned in Section 1, indeed, the service lives are on the order of several years at least. For WBES without heating, however, the membrane needs replacement every 12–15 months if the energy-storage cycle repeats once a day as proposed in Section 2.1. When the thermal load is applied, the membrane needs replacement at least twice a year. Regarding this, it should be noted that the service lives in Table 1 are meant to reveal how $N_{\max}$ varies with the thermal load (i.e., $T_{t}$). The values of $N_{\max}$ are not the focus here because practically, $N_{\max}$ can be increased by modifying the balloon. For example, the balloons can be prepared using membranes with a suitable filler [64]. Alternatively, the membranes can be tailored so that they are thicker wherever a uniformly thick membrane is more likely to start failing. In this study, however, the thinnest membranes available to the authors are purposefully chosen to expedite the fatigue failure. This restricts the durations of the experiments to hours or days instead of weeks, thus revealing the relation between $N_{\max}$ and $T_{t}$ within an affordable time frame.

### 3.2. Evolution of P–V Curves

**Figure 6:**
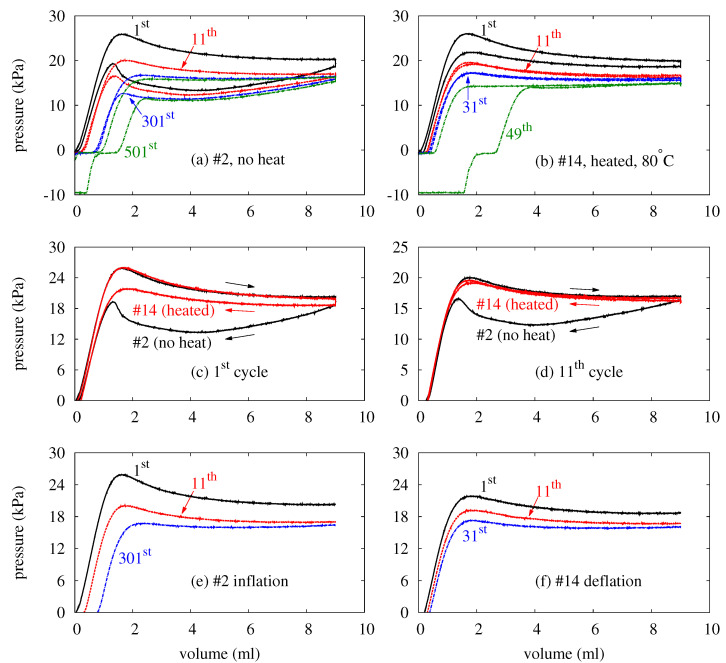
(Color online) Pressure–volume (P–V) curves of balloon #2 (no heat) and balloon #14 (heated, $T_{t}=80{}^{\circ}$C). (**a**) Four selected cycles of balloon #2. (**b**) Four selected cycles of balloon #14. (**c**) The first cycles of both balloons. (**d**) The 11th cycles of both balloons. (**e**) Three inflation curves of balloon #2. (**f**) Three deflation curves of balloon #14.

The P–V curves of balloons evolve as the loads repeat. As an illustration, the P–V curves of four selected cycles of balloon #2 (no heat) and balloon #14 (heated, 80 °C) are plotted in Figure 6a,b. These two balloons are chosen because their P–V curves are nearly identical during their first 11 inflations. The resemblence of the inflation curves facilitates the illustration of how balloons respond to heat through direct comparison of the deflation curves.

The results in Figure 6a,b reveal several important facts about water balloon energy storage. First of all, heating alters a balloon’s response to the mechanical load. This is manifest in the fact that balloon #2 and balloon #14 deflate differently ever since their first cycles. To contrast the responses of the balloons, Figure 6 overlays the P–V curves of their first cycles in cand those of their 11th cycles in d. For both cycles (and the cycles in between), heating helps balloon #14 maintain a higher pressure than that of balloon #2 at the same volume during its deflation. This reduces the area between the inflation and deflation curves and recovers a large proportion of the input energy that would otherwise be wasted.

Second, heating shortens a balloon’s service life, as discussed in Section 3.1. In Figure 6a, balloon #2 does not respond with a negative pressure (i.e., fail) until its 501st cycle. In Figure 6b, however, balloon #14 fails during its 49th cycle. These results suggest that heating can effectively reduce the service life of a balloon by one order of magnitude. This is undesirable for an energy storage system.

Despite the differences just mentioned, the P–V curves of balloon #2 and balloon #14 do have common features. To illustrate these features, Figure 6e replots three inflation curves of Figure 6a, and Figure 6f does three deflation curves of Figure 6b. In Figure 6a,b, the deflation process never returns to where the inflation of the same cycle starts. Instead, each cycle leaves a *dead volume* below which the balloon possesses negligible pressure and stores no energy. In fact, each cycle contributes a small increment to the dead volume. As such, the dead volume increases as the cycle repeats. This is manifest from the inflation curves in Figure 6e and the deflation curves in Figure 6f. In a later cycle, balloon #2 (Figure 6e) requires a larger volume to start building up its pressure. On the other hand, the pressure of balloon #14 (Figure 6f) vanishes at larger and larger volumes as the cycle repeats. By raising the dead volume, the repetition of the cycle shifts the inflation and deflation curves of one cycle to the right of those in the previous cycles. Besides the right-ward shift, the P–V curves also migrate downwards. That is, the inflation curve of each cycle lies below those of all previous cycles: in Figure 6e, the inflation curve of the 11th cycle lies below that of the first cycle, and the inflation curve of the 301st cycle is beneath those of both the first and the 11th cycles. This is also the case for each deflation curve according to Figure 6f. These features are common to all balloons in this study. On a pressure–volume diagram like Figure 6, therefore, the responses of the balloons only evolve towards the lower right. Accordingly, a balloon accommodates less and less energy as the cycle repeats.

### 3.3. Transferred Energy

For each balloon in this study, Table 1 reports the total energies that the balloon absorbs ($W_{i}$) and delivers ($W_{o}$) throughout its service life. Depending on the temperature $T_{t}$, $W_{i}$ and $W_{o}$ vary between several Joules to tens of Joules. These energies may seem small enough to render WBES completely useless. However, the energies can be readily scaled up in many different ways. For example, thicker and stiffer membranes can be adopted to implement balloons of the same base radius. Alternatively, the same membrane can be used to implement an array of balloons of a smaller base radius. With these changes, a WBES system would possess a higher pressure and can store more energy with the same amount of water.

Heating reduces $W_{i}$ and $W_{o}$, as the data in Table 1 manifest. To find the cause of the decrease, one should examine how the absorbed ($W_{ij}$) and delivered ($W_{oj}$) energies evolve and accumulate. As mentioned in Section 2.2, $W_{ij}$ and $W_{oj}$ are scaled by $W_{i1}$ so that their evolutions can be compared between different balloons in terms of $W_{ij}^{*}$ and $W_{oj}^{*}$. The energy $W_{i1}$ is chosen for the scaling because it is based on the data before a balloon is heated for the first time. For different balloons, $W_{i1}$ conveys the difference between their assemblies without any effect of the thermal load. Scaling $W_{ij}$ and $W_{oj}$ by $W_{i1}$ thus preserves the effects of heating upon these energies. As shown in Table 1, $W_{i1}$ varies between 0.174 J and 0.209 J, and the variation appears to be independent of $T_{t}$. Recall that each balloon in this study is assembled by clamping a membrane around the end of a tube. The stress for clamping a membrane is not precisely controlled, and the membrane of each newly assembled balloon warps slightly and differently. The different extents of warping then differentiate the pressures of the balloons and cause the ±9% variation of $W_{i1}$.

**Figure 7:**
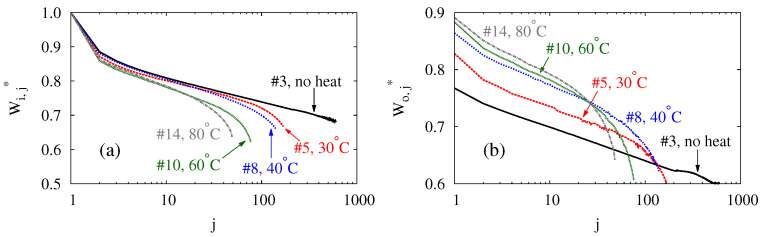
(Color online) The evolutions of (**a**) normalized input energy $W_{ij}^{*}$ and (**b**) normalized output energy $W_{oj}^{*}$ of balloons with the number of cycle *j*.

In terms of $W_{ij}^{*}$ and $W_{oj}^{*}$, Figure 7 compares the evolutions of the transferred energies between balloons subject to different thermal loads. For each thermal load, the data pertain to the balloon with the longest service life, c.f. Table 1. Only these data are plotted to avoid cluttering. According to Figure 7, balloons accommodate less and less energy as the cycle repeats. This is consistent with the observations from Figure 6, in which P–V curves evolve towards the lower right. For energy storage, the decays in $W_{ij}^{*}$ and (especially) $W_{oj}^{*}$ indicate a capacity reduction, which is undesirable. Two factors possibly underlie the decays, both are related to the Mullins effect [65,66]. First of all, the cyclic mechanical load increases the balloon’s dead volume. Second, the inflation very likely damages the membrane on the microscopic scale. As the damage accumulates, it weakens the membrane and reduces the balloon’s pressure. As discussed in Figure 6, these factors shift the P–V curves to the lower right as the cycle repeats. Admittedly, the microscopic damage is speculated but has not been examined in this study. However, the speculation seems consistent with the experimental observations. Inflating a balloon stretches its membrane, and the damage should presumably increase with the stretch. Accordingly, the apex of a balloon is the balloon’s most vulnerable spot as it endures the largest stretch [2,3,67]. In experiments, indeed, the neighborhood of the apex is where all balloons are observed to leak at the end of their service lives.

The results in Figure 7 also reveals how heating reduces $W_{i}$ and $W_{o}$ through its impact over $W_{ij}^{*}$ and $W_{oj}^{*}$. According to Figure 7a, a higher $T_{t}$ causes a faster decay of $W_{ij}^{*}$. For example, $W_{ij}^{*}$ stays above 0.7 until the 370th cycle when no thermal load is applied. When the balloon is heated with $T_{t}=30{}^{\circ}$C, $W_{ij}^{*}$ decays faster and drops below 0.7 in the 132nd cycle. Raising $T_{t}$ to 40°C makes $W_{ij}^{*}$ reach 0.7 in the 100th cycle. With $T_{t}=60{}^{\circ}$C, $W_{ij}^{*}=0.7$ occurs in 49 cycles. With 80°C, finally, $W_{ij}^{*}$ reaches 0.7 in the 35th cycle. In addition to accelerating the decay of $W_{ij}^{*}$, heating also shortens the service life. Because heating makes a balloon absorb less energy in every cycle and survive fewer cycles, a lower $W_{i}$ at a higher $T_{t}$ is a natural consequence.

Heating reduces $W_{o}$ in a similar way to how it influences $W_{i}$, although it changes $W_{ij}^{*}$ and $W_{oj}^{*}$ differently. According to Figure 7b, heating raises $W_{oj}^{*}$ by several percent during the first 20 cycles. This reflects the situation in Figure 6c,d, where the heating recovers more of the input energy. However, heating can reduce the service life up to an order depending on $T_{t}$, thereby imposing a larger sanction on the accumulation of $W_{o}$. Much like $W_{i}$, $W_{o}$ is decreased by heating because of the balloon’s shortened service life.

According to Figure 7, heating seems to influence the transferred energies differently during different stages of a balloon’s service life. For both $W_{ij}^{*}$ and $W_{oj}^{*}$, heating does not seem to exacerbate the decay significantly during the first 20 cycles. This is manifest from the similar slopes of the data in each plot during these cycles. Accordingly, the decay of $W_{ij}^{*}$ and $W_{oj}^{*}$ are independent of the thermal load during the early stage of a balloon’s service life. Subsequently, the presence of a thermal load leads to faster decays in energies, and a higher $T_{t}$ makes the decays occur earlier. These effects are more noticeable from Figure 7b. Without being heated, balloon #3 shows no clear sign of significant slope change in its data throughout its service life. For the heated balloons, the faster decay starts around the 80th cycle for $T_{t}=30{}^{\circ}$C, the 60th cycle for $T_{t}=40{}^{\circ}$C, the 30th cycle for $T_{t}=60{}^{\circ}$C, and the 20th cycle for $T_{t}=80{}^{\circ}$C. Similar trends of decays can also be found in Figure 7a for $W_{ij}^{*}$. The softening of a balloon’s membrane underlies the faster decays of $W_{ij}^{*}$ and $W_{oj}^{*}$. The softening is expected to result from the accumulation of the microscopic damage in the membrane speculated previously. To keep a balloon’s energy-storage capacity, one should adopt a rubber with a better resistance to crack growth. Alternatively, one can tailor the thickness profile of the membrane to re-distribute the stress and stretch more evenly. These modifications shall strengthen the membrane, mitigate crack growth, and retard the decays of the energies.

### 3.4. Efficiency

**Figure 8:**
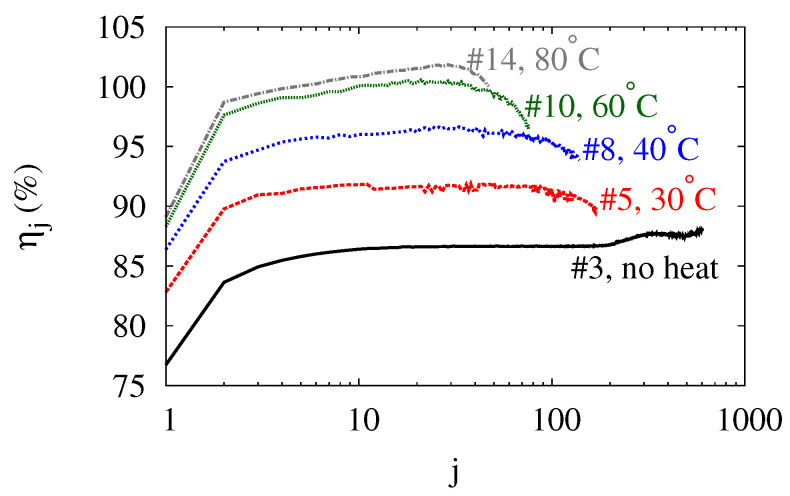
(Color online) Evolution of the round-trip efficiency $\eta_{j}$ with the number of cycle *j*.

The high efficiency is the most attractive feature of WBES. According to Table 1, the average efficiency η varies between 81% and 100%. With waste heat as low-grade as 30°C, the efficiency can be pushed beyond 90%. The round-trip efficiency $\eta_{i}$ underlies the attractive values of η, and Figure 8 shows the evolutions of $\eta_{i}$ for balloons subject to different thermal loads. For each balloon, $\eta_{i}$ increases drastically in the second cycle. Then it persists until close to the end of the balloon’s service life. Without the help of heating, the efficiency varies between 85%–90%. Heating improves the efficiency of WBES, and the improvement depends on the temperature $T_{t}$. With $T_{t}=30{}^{\circ}$C, $\eta_{i}$ stays around 90%. Raising $T_{t}$ to 40°C increases $\eta_{i}$ by 5%. Further raising $T_{t}$ to 60°C raises the efficiency by another 5%, pushing $\eta_{i}$ close to 100%. In that case, the water balloon recovers almost all the work that the water does to inflate it. Finally, adopting $T_{t}=80{}^{\circ}$C enables the balloon to achieve $\eta_{i}$ beyond 100%. These results suggest that a water balloon can effectively utilize low-grade waste heat to boost its efficiency. To avoid any confusion, it should be emphasized that the efficiencies do not account for the cost of the low-grade heat. With the heat being freely available, an efficiency beyond 100% becomes possible and does not violate the conservation of energy.

### 3.5. Permanent Deformation

As discussed with Figure 6, the cyclic mechanical load creates a dead volume that increases monotonically as the cycle repeats. From an engineering perspective, the dead volume is undesirable as it keeps no pressure, stores no energy, and degrades the balloon’s performance. Scientifically, however, it is interesting to note that the dead volume seems to approach the volume of a hemisphere with the same base radius as that of the balloons. This is most clearly shown by comparing the balloon’s base radius $r_{b}$ to its average radius of curvature $R_{a}$ corresponding to the dead volume $V_{d}$. The radius $R_{a}$ is the radius of curvature of a spherical cap with base radius $r_{b}$ and volume $V_{d}$. The parameters $R_{a}$, $r_{b}$ and $V_{d}$ are related by

(6) $$V_{d}=\frac{\pi }{3}\left[2R_{a}^{3}-\left(2R_{a}^{2}-r_{b}^{2}\right)\sqrt{R_{a}^{2}-r_{b}^{2}}\right]$$

**Figure 9:**
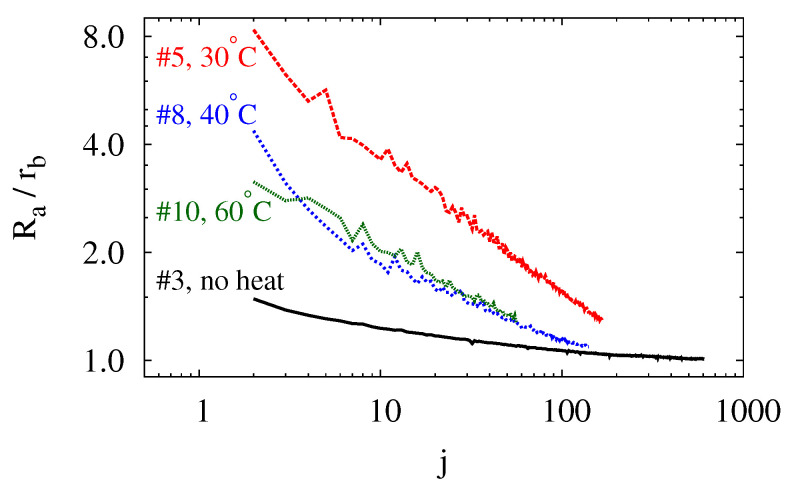
(Color online) Evolution of the average radius of curvature of a balloon’s permanent deformation.

In Figure 9, the ratio of $R_{a}$ to $r_{b}$ is plotted against the number of cycles for most of the balloons in Figure 7 and Figure 8a. To avoid cluttering, the curve for balloon #14 ($T_{t}=80{}^{\circ}$C) is not plotted because it lies right between those of balloon #8 and #10. For each balloon in Figure 9, $R_{a}$ exhibits an overall decreasing trend with some occasional fluctuations. Additionally, $R_{a}$ decreases at more and more gentle rates as the cycle repeats. The heated balloons fail relatively early before their $R_{a}/r_{b}$ ratios have a chance to exhibit a clear lower bound. For balloon #3, which is not heated, $R_{a}$ ends up becoming $1.003r_{b}$. Based on these, $r_{b}$ seems to be the lower limit that $R_{a}$ ‘strives’ to approach over the several hundred cycles. For spherical caps with the same base radius, a hemisphere is the one with the smallest radius of curvature. The observation apparently suggests that a pinned circular membrane evolves to approximate a hemisphere and minimize its radius of curvature when it is subject to cyclic mechanical load.

## 4. Conclusions

This study proposes the use of water balloons as an energy storage medium and tests their performances experimentally. In each experiment, a water balloon is subject to cyclic mechanical and thermal loads, and its response is examined in terms of their pressures, transferred energies, efficiencies, and service lives. With the results already presented, a first contribution of this study is the discovery of the excellent efficiencies that a water balloon achieves as an energy storage medium. A second contribution is the demonstration of recycling low-grade waste heat with a water balloon. Finally, a third contribution is the disclosure of the trade-off between the efficiency and the service life of a balloon. Candidate solutions are also proposed to enlarge the capacity and extend the service life of a balloon when it serves as an energy storage medium. These results shall serve as a useful guide for developing balloon-based thermo-mechanical devices in a broad range of applications including but not limited to the storage of energy.

## Figures and Tables

**Table 1:** The parameters of all experiments in this study. For each balloon, $T_{t}$ is the thermal load temperature of its heating process, $T_{m}$ and $\sigma_{T}$ are the average ($T_{m}$) and the standard deviation ($\sigma_{T}$) of the thermal bath temperature during the deflation process, $N_{\max}$ is the service life, $W_{i}$ and $W_{o}$ are the total energies it absorbs ($W_{i}$) and delivers ($W_{o}$) throughout its service life, $W_{i1}$ is the energy it absorbs during its first inflation, and η is the average efficiency.

ID	$T_{t}$	$T_{m}$	$\sigma_{T}$	$N_{\max}$	$W_{i}$	$W_{o}$	$W_{i1}$	η
	°C	°C	°C	Cycles	J	J	J	%
1	RT	RT	–	388	51.87	44.07	0.183	84.95
2	RT	RT	–	501	69.95	56.79	0.195	81.18
3	RT	RT	–	604	74.87	65.28	0.174	87.19
4	30	39.53	0.35	132	19.91	18.11	0.209	90.99
5	30	39.46	0.43	170	24.00	21.87	0.194	91.10
6	30	39.54	0.58	163	22.75	20.92	0.188	91.98
7	40	49.65	0.35	103	14.51	13.78	0.193	94.92
8	40	49.01	0.36	140	19.46	18.58	0.190	95.48
9	40	48.38	0.31	88	13.27	12.59	0.199	94.87
10	60	66.38	0.37	76	10.87	10.59	0.196	97.46
11	60	66.79	0.31	56	7.50	7.39	0.178	98.47
12	60	66.88	0.41	53	8.10	7.81	0.203	96.45
13	80	82.88	0.52	30	4.34	4.16	0.194	95.79
14	80	80.57	1.20	48	6.85	6.85	0.189	99.97
15	80	80.38	0.97	41	5.95	5.84	0.194	98.19

## Data Availability

The data that support the findings of this study are available within the article. Further details are available from the corresponding author upon reasonable request.
